# Design of a Lossless Image Compression System for Video Capsule Endoscopy and Its Performance in *In-Vivo* Trials

**DOI:** 10.3390/s141120779

**Published:** 2014-11-04

**Authors:** Tareq H. Khan, Khan A. Wahid

**Affiliations:** Department of Electrical and Computer Engineering, University of Saskatchewan, Saskatoon, SK S7N5A9, Canada; E-Mail: tareq.khan@usask.ca

**Keywords:** capsule endoscopy, compression system, image sensor, *in-vivo* trial

## Abstract

In this paper, a new low complexity and lossless image compression system for capsule endoscopy (CE) is presented. The compressor consists of a low-cost YEF color space converter and variable-length predictive with a combination of Golomb-Rice and unary encoding. All these components have been heavily optimized for low-power and low-cost and lossless in nature. As a result, the entire compression system does not incur any loss of image information. Unlike transform based algorithms, the compressor can be interfaced with commercial image sensors which send pixel data in raster-scan fashion that eliminates the need of having large buffer memory. The compression algorithm is capable to work with white light imaging (WLI) and narrow band imaging (NBI) with average compression ratio of 78% and 84% respectively. Finally, a complete capsule endoscopy system is developed on a single, low-power, 65-nm field programmable gate arrays (FPGA) chip. The prototype is developed using circular PCBs having a diameter of 16 mm. Several *in-vivo* and *ex-vivo* trials using pig's intestine have been conducted using the prototype to validate the performance of the proposed lossless compression algorithm. The results show that, compared with all other existing works, the proposed algorithm offers a solution to wireless capsule endoscopy with lossless and yet acceptable level of compression.

## 1. Introduction

Capsule endoscopy (CE) [1,2] is a non-invasive technique to receive images of the intestine for medical diagnostics. The main design challenges of endoscopy capsule are acquiring and transmitting acceptable quality images by utilizing as little hardware and battery power as possible. In order to save wireless transmission power and bandwidth, an image compressor needs to be implemented inside an endoscopy capsule. Lossy image compressors produce some difference between the original and reconstructed images. For medical diagnostics, the distortion of the reconstructed image can lead to inaccurate diagnostics decisions, though in medical and endoscopic imaging, lossy compression is acceptable up to a certain point (for example, a compression ratio of 15 was found as the visually lossless threshold for the JPEG lossy algorithm [3]). However, in hospitals in these days where Picture Archiving and Communication Systems (PACS) are used to store medical and diagnostic data in digital form, lossless compression is a requirement [4]. In addition, lossless compressors produce identical reconstructed images compared with the original images without any distortion. Therefore, the prime objective has always been to find efficient and lossless compression methods.

In this paper, a low complexity yet lossless image compression algorithm is presented that is purposely designed for capsule endoscopy. The compressor works on a highly efficient YEF color space [5], which is specially designed to compress endoscopic images by analyzing the unique image properties. After color space conversion, the compressor takes the difference of consecutive pixels using differential pulse coded modulation (DPCM) and then encodes the differences in variable length Golomb-Rice [6] and unary coding. A customized corner clipping scheme is also implemented to remove uninteresting corner area of the image to increase compression ratio. All these components are fully optimized for low-cost operation and lossless in nature; as a result, no loss of any diagnostic information takes place inside the endoscopic system.

In order to validate the performance of the compression algorithm, it is deployed inside an endoscopic capsule prototype developed in our lab. At first, a modular and programmable CE development system platform consisting of a miniature field programmable gate array (FPGA) based electronic capsule is developed. The prototype supports various imaging modes including the commonly used white light imaging (WLI) and narrow band imaging (NBI) [7], and communicates with the data logger in full duplex fashion, which enables configuring the image size and imaging mode in real time during the examination. The CE prototype is then tested to assess the performance using live pig in both *ex-vivo* and *in-vivo* trials at the animal facility.

## 2. Related Works

Both lossy and lossless image compression algorithms are found in the literature targeting capsule endoscopy application. A brief discussion on both types of compression is given below.

### 2.1. Lossy Compression Algorithms

**Figure 1.**
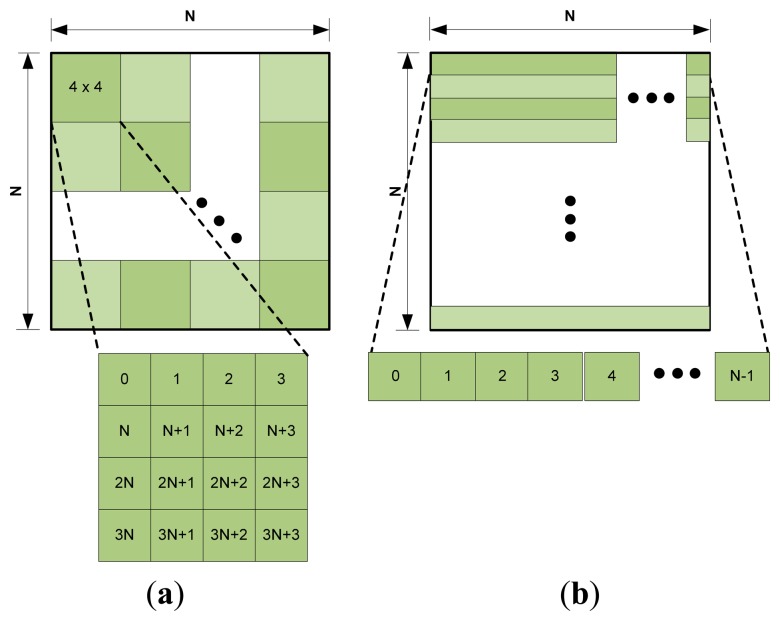
Pixel access sequence in image sensor using two topologies: (**a**) 4 × 4 block; (**b**) raster-scan.

The lossy algorithms found in literature are mainly based on transform coding where the Discrete Cosine Transform (DCT) is used [8–15]. In these algorithms, image pixels need to be accessed in 4 × 4 or 8 × 8 blocks from the image sensor as shown in Figure 1a. However, commercially available complementary metal-oxide-semiconductor (CMOS) image sensors [16,17] send pixels in raster scan fashion (*i.e.*, row-by-row, from top left to right) as shown in Figure 1b. These image sensors also do not have internal buffer memory for image storage and random access of pixels. Due to the mismatch of pixel steaming sequence of commercial image sensors and pixel access sequence required by transform based compression algorithms, buffer memory needs to be implemented inside the capsule to store a complete or blocks of an image frame, so that the image pixels can be accessed by the compressor in block wise fashion from the buffer memory.

In order to start processing of the first n × n block of an N × N size image, the compressor has to wait and store all the pixels coming from the image sensor to buffer memory until the first n × n block is available. The memory requirement in bits, *S*, can be expressed as Equation (1):

(1) S=(N×(n−1)+n)×BPP

where *BPP* is the bits-per-pixel. For 320 × 320 image size and 8 × 8 block access of 24 BPP pixels, 6.6 kB buffer memory need to be implemented inside the capsule. Moreover, when the compressor will start working with the 8 × 8 block pixels, the image sensor will continue to send its remaining pixels and more memory will be required to store those pixels while the compressor is busy. One solution of this problem is to pause the image sensor after it sends all the pixels for accessing a block; however the feature of pausing an image sensor in the middle of transmitting pixels of a frame is not found due to the read out timing requirements of the pixel array in the image sensors. Another possible solution to the problem is to use two buffer memory of total size 2*S*, so that while the compressor works with pixels of one buffer, the new pixels continuously coming from the image sensor are stored in the other buffer. However, this solution can create timing error if compression and transmission time exceeds the input data rate of the image sensor. A safer solution of this problem could be to use sufficient memory to store a complete image frame. For storing a 320 × 320 size image of 24 BPP, 300 kB memory is required. Buffer memory takes significantly large silicon area and consumes sufficient amount of power which can be a noticeable overhead in capsule endoscopy application. Moreover, complex calculations are associated with transform coding based compressors (*i.e.*, multiplications, additions, data scheduling, *etc.*), as a result large silicon area and power consumption are required which makes them less suitable for practical implementation.

The works in [5,18] by our group present lossy compression algorithms which do not require block based access of image pixels; rather they can work with pixels coming in raster scan fashion. However, these compressors (both raster scan based and block based) presented in the literature are lossy compressors which incur various levels of distortions in the reconstructed images. For medical diagnostics, these distortions can lead to inaccurate diagnostics decisions. Moreover, these works present only computer based simulation results using endoscopic images taken from on-line databases (such as [19]). The performance of their algorithms was not validated using hardware level simulation or *ex-vivo* trials.

### 2.2. Lossless Compression Algorithms

While lossy compression algorithms for capsule endoscopy are in abundance, their lossless counterpart is only a few. In [20], our group proposed a lossless image compressor based on YUV color space. However, YUV color space is computationally expensive due to the presence of floating point numbers during conversion from RGB and it consumes significant area and power when implemented in hardware. Besides, the practical limitation of the work is that the architecture assumed that the image sensor must have built-in RGB to YUV color space converter which limits the robustness. Moreover, the performance was validated using only image simulation without any deployment into hardware platform or any capsule endoscopic system. Another choice for lossless compression is to use the JPEG-LS algorithm [21–23]. No work was found that uses JPEG-LS for capsule endoscopy application; as a result, we have implemented it and applied to our dataset. The results are added later in this paper. Lastly, the work in [24] proposed a modified JPEG-LS algorithm for endoscopic image compression which is not entirely lossless. The work provides simulation results only without any *in-vivo* trial for performance validation.

In contrast, here the proposed lossless compressor can work with any elementary RGB based image sensor that outputs image pixels in raster scan fashion which eliminates the need of a memory buffer. The compressor consists of a low complexity color space, known as YEF, designed by analyzing the unique properties of endoscopic images [5]. As the compressor is lossless, it produces reconstructed images without any distortion and thereby reduces the possibility for inaccurate diagnostics. The performance is validated using a complete CE system developed in our lab with *ex-vivo* trials with live pig.

## 3. The Lossless Image Compressor

### 3.1. Design Requirements

While designing the lossless compression algorithm, we have set the following design objectives:

- Low power consumption
  The endoscopic capsule runs on button batteries. So, the compression algorithm should be of low complexity and consume low power when implemented in hardware.
- Less silicon area
  The size of the capsule should be as small as possible. Memory consumes significant silicon area and power. So, we focus on algorithms that require less memory.
- Interfacing with commercial image sensors
  Commercially available CMOS image sensors [16,17] send pixel data in raster-scan fashion. So, the compressor should be able to accept input pixels coming in raster scan fashion which will make it compatible with commercial image sensors.
- High compression ratio
  The lossless algorithm must be able to reduce the data to be sent by the transmitter in order to fit into the bandwidth of the transceiver and to save transmission power.
- Support for multiple imaging modes
  The compression algorithm should support various imaging modes, such as WLI and NBI, and should equally produce high compression ratio in both cases.

### 3.2. Proposed Lossless Algorithm

**Figure 2.**
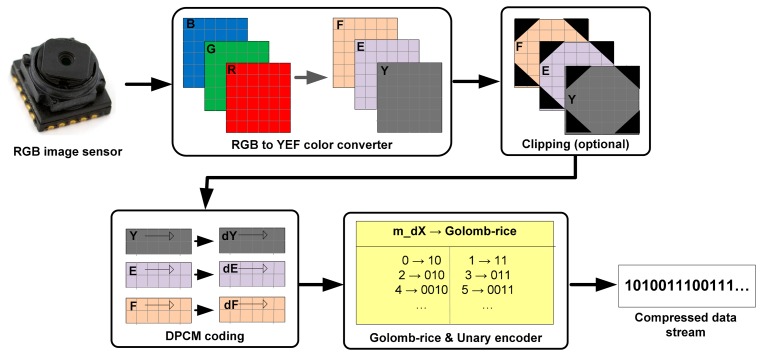
Block diagram of the proposed lossless compression algorithm.

The block diagram of the proposed lossless compression algorithm is shown in Figure 2. At first, the RGB pixels are converted to YEF color space. Then an optional clipping module is added that removes uninteresting corner area of the image.

A lossless predictive encoder, known as differential pulse coded modulation (DPCM) is used. The differential values of luminance component are encoded in Golomb-Rice code [6] where the differential values of chrominance components are encoded in unary code. The different stages of the proposed algorithm as placed in the processing pipeline are briefly discussed below:

#### 3.2.1. RGB to YEF Conversion

At this first stage of the algorithm, RGB pixels are converted to YEF color space [5], which is suitable for CE image compression and efficient for hardware implementation. The color space is designed by analyzing the unique properties of endoscopic images for better compression. The motivation for the YEF color space comes from the fact that, endoscopic images generally exhibit dominance in red color with the absence of significant green and blue components. Our experiments show that, in most cases, the intensity distribution of green in endoscopic images is very similar to that of blue component. Experiments have also shown that the intensity distribution of luminance (Y) has similar pattern of green and blue components – thus, subtracting green and blue components form the luminance will produce differential pixel values of almost equal numbers and will reduce the entropy of the chrominance planes. Reduced entropy will cause higher compression ratio in the chrominance planes. Conventional color spaces such as YUV and YCoCg do not exhibit such properties.

In YEF, the luminance is stored in Y component, E stores the difference between luminance and green component, and F stores the difference between luminance and blue component. The relationships are shown in Equations (2)–(4).

(2) $$Y=\frac{R}{4}+\frac{G}{2}+\frac{B}{4}$$

(3) $$E=\frac{Y}{2}-\frac{G}{2}+128=\frac{R}{8}-\frac{G}{4}+\frac{B}{8}+128$$

(4) $$F=\frac{Y}{2}-\left(\frac{3B}{8}+\frac{G}{8}\right)+128=\frac{R}{8}+\frac{G}{8}-\frac{B}{4}+128$$

**Figure 3.**
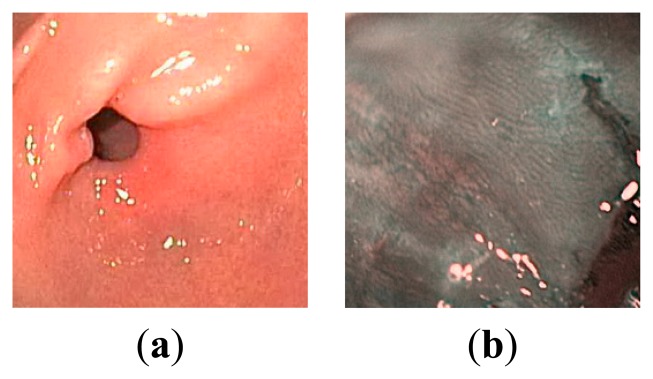
Sample endoscopy images: (**a**) WLI; (**b**) NBI.

**Figure 4.**
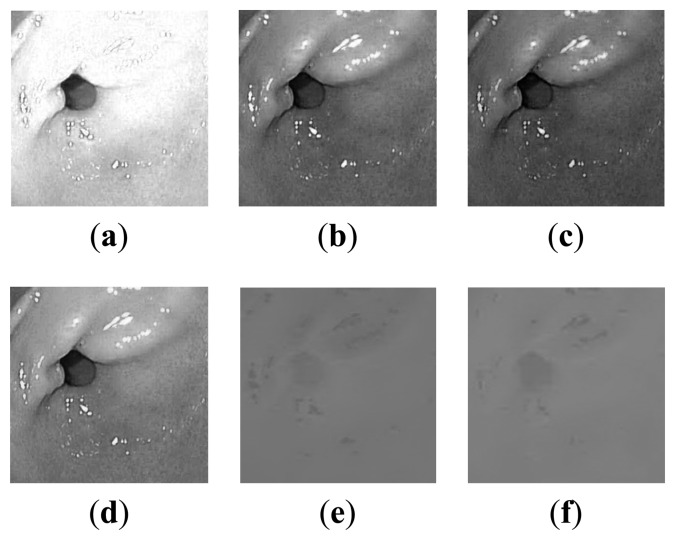
Intensity distribution of color components of a WLI image: (**a**) R, entropy: 6.15; (**b**) G, entropy: 6.74; (**c**) B, entropy: 6.53; (**d**) Y, entropy: 6.65; (**e**) E, entropy: 3.44; (**f**) F, entropy: 3.26.

**Figure 5.**
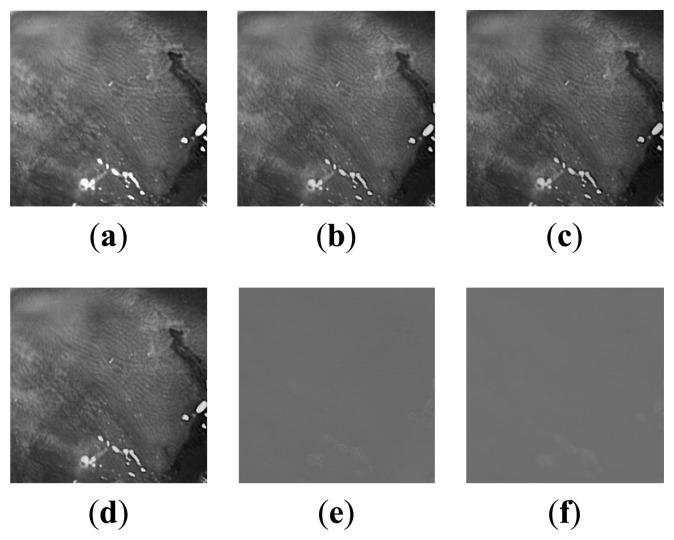
Intensity distribution of color components of an NBI image: (**a**) R, entropy: 6.80; (**b**) G, entropy: 6.73; (**c**) B, entropy: 6.68; (**d**) Y, entropy: 6.73; (**e**) E, entropy: 2.14; (**f**) F, entropy: 1.94.

In Figure 3, two standard WLI and NBI endoscopic images are shown, taken from [19]. In Figure 4, the plots of all color component values of the WLI image (Figure 3a) are shown in grayscale with entropy (bits/pixel) mentioned. It can be seen from Figure 4 that, after converting to YEF, there is less change in pixel values in chrominance (E and F) components of YEF color space than RGB components, which indicates that less information or entropy is contained there and these two components can be compressed heavily. In Figure 5, the intensity distributions for the NBI image (Figure 3b) are shown and they also reveal that the chrominance components of YEF color space contain low information content or entropy.

Note that, the YEF color space does not discard the chrominance information; in fact, it is another representation of the RGB color space which is more suitable for compression and theoretically lossless. The YEF color components can be converted back to RGB color components using Equations (5)–(7):

(5) R=Y+3.33×(E−128)+2.67×(F−128)

(6) G=Y−2×(E−128)

(7) B=Y+0.67×(E−128)−2.67×(F−128)

From Equations (2)–(4), it is observed that the conversion between color spaces involves only a few additions and divisions by numbers that are powers of 2, which can be implemented by shift operations in digital hardware. When Equations (2)–(4) are implemented in digital hardware as integers, minor variations in the pixel values may occur due to the rounding of fractions to integers. The YEF color space can be made fully reversible (*i.e.*, reconstructed image is statistically identical with original image) by adding 3 more extra bits for storing fraction along with the 8 bits for integer. The average image quality of 100 endoscopic images (taken from [19]) are shown in Table 1 where the pixels are first converted from RGB to YEF and then converted back to RGB. It can be seen that for a bit precision of 11-bits, the conversion is lossless (or 100% reversible).

#### 3.2.2. Corner Clipping

In capsule endoscopy, the corner areas in a captured image are often blacked out. To achieve it, an optional corner clipping algorithm can be employed (as described in [20]) during the image acquisition stage.

#### 3.2.3. Differential Pulse Coded Modulation (DPCM)

**Figure 6.**
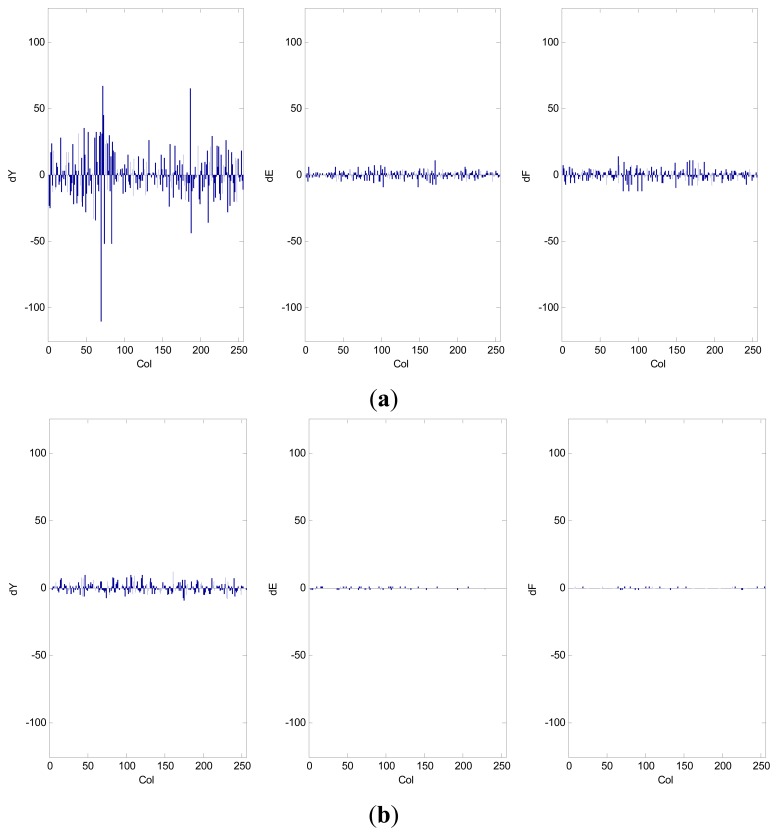
Change in consecutive pixel value at row 128 of (**a**) standard 256 × 256 “mandrill” image; (**b**) 256 × 256 WLI endoscopy image.

In this stage, a lossless predictive coder is used. Due to the rare occurrence of sharp edges in endoscopic images, the difference between the component values of two consecutive pixels is generally small. The change in component values (*dX*) with respect to its adjacent left pixel in any row is given by Equation (8):

(8) $$dX_{r.c}=X_{r,c}-X_{r,c-1}$$

where, *X _r,c_* is the pixel value at row *r* and column *c*, and *X _r,c-1_* is its adjacent left pixel value. *X* can represent Y, E, or F component values. Figure 6 shows the changes in *dX* for a standard “mandrill” image and as well as for an endoscopic image. From Figure 6, it is seen that smaller changes in pixels values occur in endoscopic images. Similar phenomenon is observed for other endoscopic images used in this work.

More simulations have been conducted with 100 WLI and 15 NBI test endoscopy images and with standard images; average absolute difference (AAD) is used as given in Equation (9) as the statistical measure of *dX*:

(9) $$dX_{\text{avg},\text{image}}=\frac{\overset{N}{\underset{n=1}{\text{∑}}}\overset{M-1}{\underset{m=1}{\text{∑}}}\left|\left(x_{m+1,n}-x_{m,n}\right)\right|}{M\times N}$$

where, *M* and *N* are the image width and height namely; *x* is the original component value. The results are summarized in Table 2, where it is seen that, in general, the difference in pixel (*dX*) with respect to the adjacent left pixel is very small in endoscopic images compared to that of standard images. As a result, the DPCM is a good choice. It should be noted that, the DPCM used here does not use any quantization and thus is lossless as well. Besides, it has very low computational complexity which will help reduce power and area consumption of the compressor.

#### 3.2.4. Variable Length Coding

The difference of the consecutive pixels (*dX*) is then mapped to a non-negative integer and then they are encoded in variable length coding. To get the best compression ratio, the difference of luminance (*dY*) is encoded in Golomb-Rice code and the difference of chrominance (*dE* and *dF*) are encoded in unary for WLI images. In all cases of luminance and chrominance, *dX* is mapped one-to-one with another integer set, *m_dX* and divided by a predefined integer, 2*^k^*. In Golomb-Rice coding, the choice of *k* parameter is important since it dictates the code length. A detailed discussion on choosing *k* parameter can be found in Section 3.3.1 of [20]. The unary code can be generated by setting the Golomb-Rice code parameter *k = 0* in the encoder. For NBI, *dY* generally spans wider than WLI due to the presence of sharper edges in NBI images. This phenomenon can be noticed from Table 2. To get the best compression ratio, the *k* is set as 2 for WLI images and 3 for NBI images for encoding *dY*. The *k* parameter values are summarized in Table 3. Pseudo-color NBI images are reconstructed by combining two grayscale images in the computer software. One image is captured using green light and another using blue light. As the input NBI images are grayscale, only the luminance (Y) component is compressed and transmitted. The chrominance components are not sampled for NBI images and pseudo color is added later on the images using computer software.

### 3.3. Simulation Results

**Figure 7.**
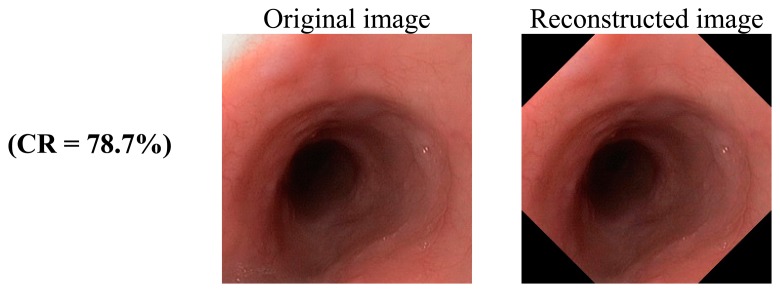

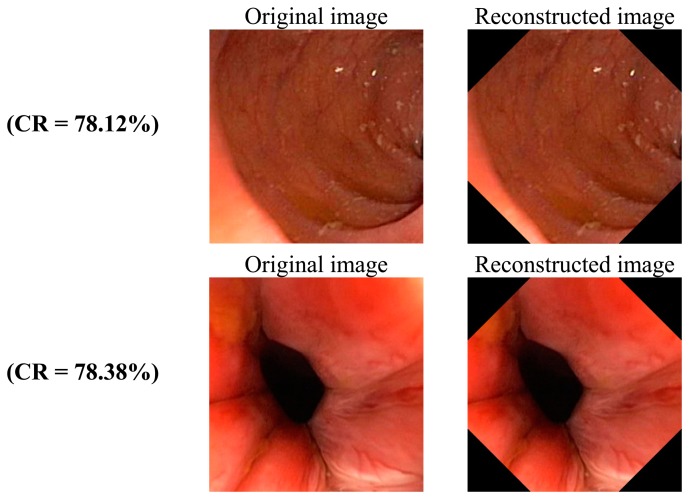
Original and reconstructed images; they are identical since the algorithm is lossless in nature; optional corner clipping is used.

As the proposed compression algorithm is lossless, the reconstructed image is identical to the original image. In Figure 7, several original and reconstructed images are shown with compression ratio (CR) mentioned. The average CR of a number of WLI endoscopic images and video frames are shown in Table 4.

From Table 4, it is found that the proposed lossless compression algorithm produces approximately 78% average compression ratio (CR) for different types of images. The CR of NBI images are also shown in Table 5. In this case, the algorithm produces an average CR of 84%.

It is noted that in our implementation (also in Tables 4 and 5), we have used integer precision with 8-bits. As mentioned before for Equations (2)–(4), the integer division in the RGB-YEF conversion discards the fraction bits. It may generate very small variations in pixel values at the output stream which is visually unnoticeable to human eyes when displayed as an image. However, in order to generate theoretically reversible compressed bit stream, one may add additional bits to preserve the fractions. The average CR for different number precision bits for both WLI and NBI video sequences are shown in Table 6.

In Table 7, the proposed compressor is compared with other related compression algorithms. Here, we see that the proposed compressor outperforms in CR and in image quality when compared with [14,24]. The works in [8–10,12,13,15] are lossy compressors; these algorithms produces distortions in the reconstructed images which may lead to inaccurate diagnostics. Moreover, the algorithms presented in these works are based on DCT which has computational complexity of O(*n log n*) and need buffer memory (as described earlier). The work in [20] produces lossless reconstructed images, however, the proposed compressor has better compression ratio. We have also implemented the JPEG-LS [21] algorithm and applied it to our dataset. When comparing with JPEG-LS, the proposed algorithm has higher compression ratio, lower computational complexity (such as static prediction and static *k* parameter) and lower memory requirement. The proposed compressor works on a low complexity YEF color space and it has a computational complexity of O(*n*) with no buffer memory requirements for image storage. Besides, the works of [20,21] do not support NBI imaging mode.

In order to compare the proposed YEF color space for endoscopic images with the conventional YCoCg color space, we have conducted additional simulations by replacing the YEF color space by YCoCg in the proposed compression algorithm. Our experiments show that YCoCg color space produces a CR of 71.2% where YEF produces a higher CR (74.7%) for the same data set. Besides, the YCoCg color space generates orange and green chrominance components which have no added significance when considering the properties of endoscopic images.

## 4. Performance Evaluation in a Capsule Prototype

In order to validate the performance of the proposed lossless compression algorithm in a real-world scenario, it is employed inside a capsule endoscopy prototype. The prototype is developed in our lab that consists of three major units—the capsule, the data logger and the computer software running in desktop computer.

### 4.1. Electronic Capsule

**Figure 8.**
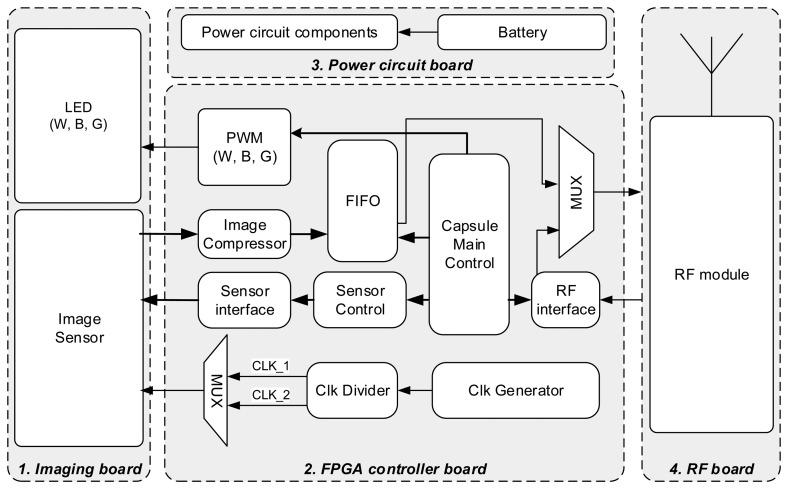
Architecture of the capsule prototype.

To make the hardware modular, the capsule is divided into four boards: Imaging board, FPGA board, RF board and Power board. The overall block diagram of the capsule is shown in Figure 8. The components were chosen considering performance, power requirement and physical size to fit into a miniature capsule prototype. The size of the capsule is 16 mm × 36 mm. However, it should be noted that the purpose of this work is to demonstrate the advantages of the proposed compression system in *in-vivo* trial; as a result, the capsule was intended to be such that can be swallowed by pig, not humans, so, its size should not be compared with a commercial capsule which is normally 11 mm × 28 mm.

The Imaging board contains a CMOS image sensor with lens [16] and four LEDs. To support both WLI and NBI imaging modes, two white LEDs, one blue LED having a peak wavelength of 405–410 nm and one green LED having a peak wavelength of 520 nm are used in the design. The FPGA board contains a Lattice FPGA MachXO2-2000 and an RC reset circuit. The FPGA chip has physical dimension of 8 × 8 × 1.23 mm and contains 2112 lookup tables (LUTs), non-volatile RAM for storing configuration and built in clock generator which makes it a suitable choice for miniature capsule endoscopy application. The proposed compression algorithm is implemented in VHDL and programmed into the FPGA along with other necessary control modules.

The RF board contains a 2.4 GHz RF transceiver by Nordic and other necessary passive components [25]. Although this wavelength shows high absorption characteristics of electromagnetic radiation of by water, previous works in [26,27] have shown that 2.4 GHz transceivers, like Nordic, can be effectively used to transmit data wirelessly through animal body. It is noted here that, medical implantable communication service (MICS) compatible RF transceivers that work at 402–405 MHz frequency, are the most suitable choice for transmitting data through human body [28]. Pillcam capsule by Given Imaging uses a custom made MICS compatible RF transceiver from Microsemi (part ZL70081 with data-rate of 2.7 Mbps [29]). However, this unit is not offered for public purchase; instead they offer a low data-rate transceiver (part ZL70102 with data rate of 500 kpbs [26]) which is not even sufficient for 2 frames per sec application. As a result, we choose to use a 2.4 GHz Nordic transceiver having a raw data-rate of 2 Mbps in our design.

Moreover, the Nordic transceiver contains cyclic redundancy check (CRC)-based error detection and retry with auto acknowledgement feature. In auto acknowledge mode, after receiving a data packet, the receiver checks the CRC bits and detects whether there was any error during the transmission of the packet. If there was any error, then it requests the transmitter to resend the data-packet again. This process goes on until the packet is transmitted successfully. So, in auto acknowledgement mode, generally no data loss happens. Experiments have been conducted to show a bit error rate (BER) of less than 0.1% [25]. The Power board contains miniature low dropout regulators (LDO), a magnetic reed switch and three silver oxide button batteries, each having a rated voltage of 1.55 V and capacity of 195 mAh.

### 4.2. Data Logger

A microcontroller based data logger is used for storing image data [30]. After logging, the image data can be viewed in real-time on the data logger's LCD screen or transferred to computer using a SD card.

### 4.3. Computer Software

The software is simply an image decoding engine that decodes the compressed images and generates viewable image data. Parameters for reconstructing NBI images can be set by the user in the decoder module.

### 4.4. Results and Discussion

**Figure 9.**
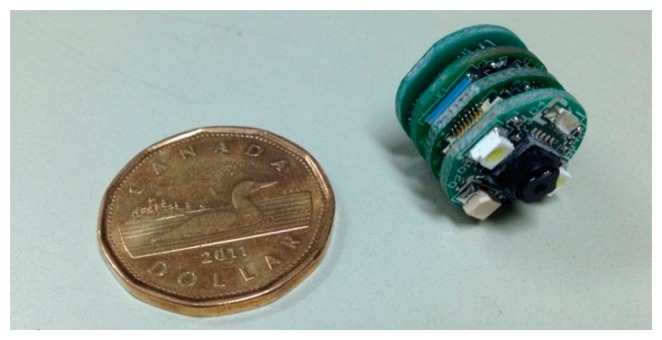
Photograph of the capsule prototype (size compared with a 26.5 mm diameter 1$ Canadian coin).

The four boards are manufactured in circular PCBs each having a diameter of 16 mm as shown in Figure 9. The digital blocks in the FPGA are modeled and synthesized in the FPGA of the capsule prototype. The synthesis results of the proposed lossless compressor are summarized in Table 8.

The measured current consumption of the capsule prototype is 23 mA and the capsule can run for 8.5 h with three 195 mAh button batteries.

### 4.5. Comparison with Other Prototype Works

In Table 9, the proposed compressor is compared with similar other works. It is noticed that the proposed design has additional support for NBI mode, does not require significant buffer memory to store image pixels and still can produce compressed bit stream without any data loss which is a key feature of the design. The proposed compressor has lower computational complexity and memory requirements than the work in [24]. However, the compressor in [24] is implemented in ASIC platform, which resulted in lower power consumption. Also, the performance of the proposed compressor has been validated in *in-vivo* and *ex-vivo* trials.

## 5. Performance Evaluation in Animal Trial

In order to validate the performance of the developed CE system, the system was tested in pig's intestine for both *ex-vivo* and *in-vivo* cases. Pig's intestine is chosen for experiment due to its relatively similar gastrointestinal functions in comparison to humans [33]. The experimental setup and results are briefly discussed below.

### 5.1. Ex-Vivo Testing

**Figure 10.**
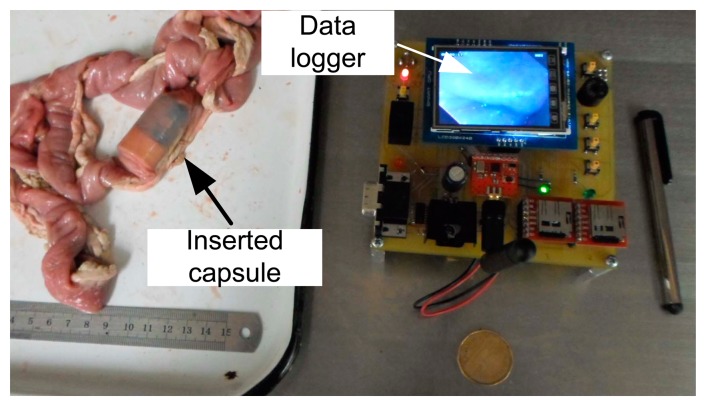
Experimental setup: capsule inserted in pig's intestine and the data logger is showing real-time inside image of the intestine.

**Figure 11.**
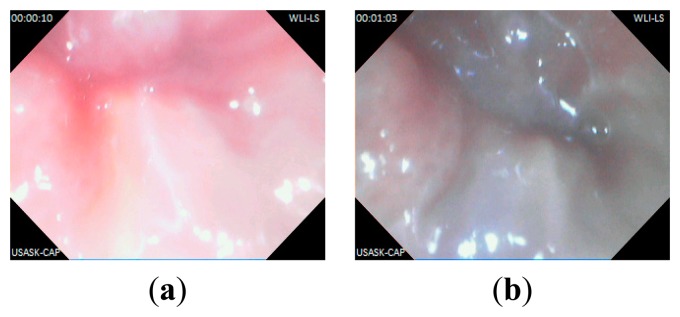
Captured lossless WLI images from pig's intestine: (**a**) CR = 79.71%; (**b**) CR = 75.61%.

**Figure 12.**
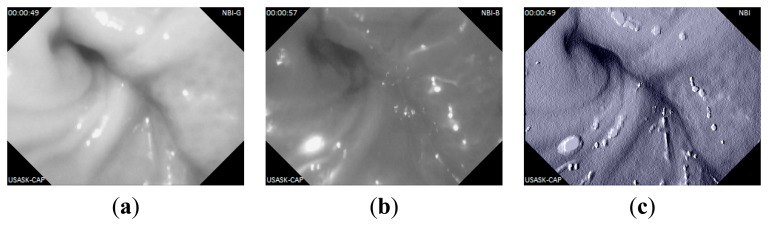
Captured NBI images from pig's intestine: (**a**) grayscale image with green light, CR = 84.31%; (**b**) grayscale image with blue light, CR = 84.11%; (**c**) Combined pseudo color NBI image from (a) and (b).

In this experiment, the capsule prototype is inserted inside a section of pig's small intestine; the data logger is placed outside. The experimental setup is shown in Figure 10. The capsule then captured images of intestine and sent data to the data logger unit. The data logger stores the image data and also shows reconstructed image on LCD. The image data are then transferred to computer for reconstruction. Several captured lossless WLI and NBI images are shown in Figures 11 and 12 namely with achieved CR. The received images have good quality and detailed features of the mucosa are visible in the images. The CR of the images in the real experiments is similar to the results found during simulation.

During the experiment, the distance between the capsule and the data logger is varied from 0.3 m to 1 m and images are transmitted successfully. As the data logger is wearable and it is generally worn at one side of the belly, the distance between a swallowed capsule and data logger will be near 0.3 m for human endoscopy.

### 5.2. In-Vivo Testing

**Figure 13.**
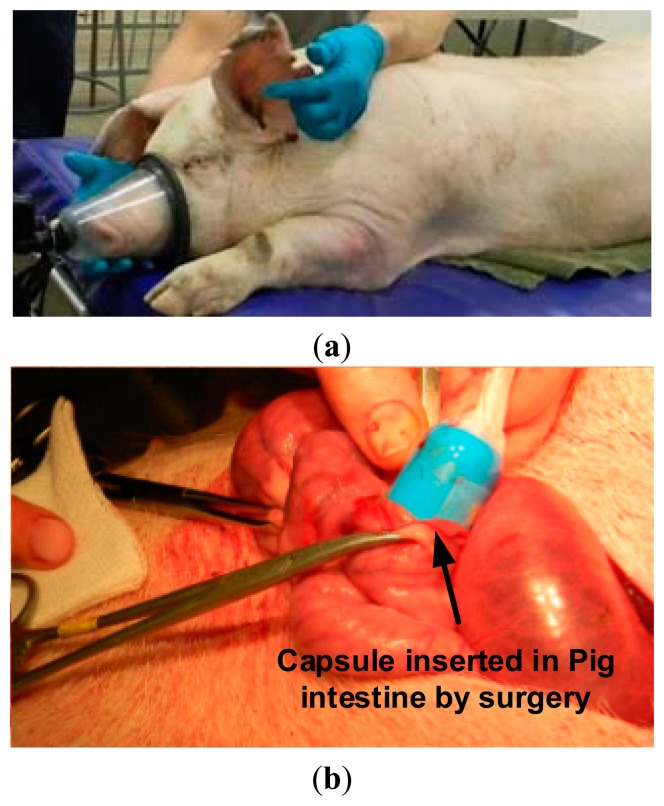
(**a**) Live pig under anesthetic at the Prairie Swine Center, Saskatoon, SK, Canada; (**b**) capsule inserted in live pig's intestine by surgery.

**Figure 14.**
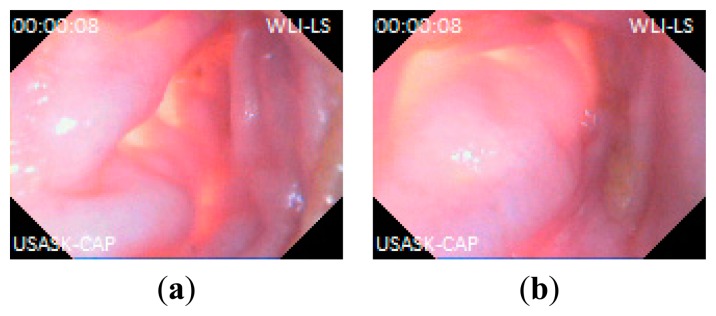
Captured lossless WLI images from live pig's intestine: (**a**) CR = 72.43%; (**b**) CR = 74.09%.

**Figure 15.**
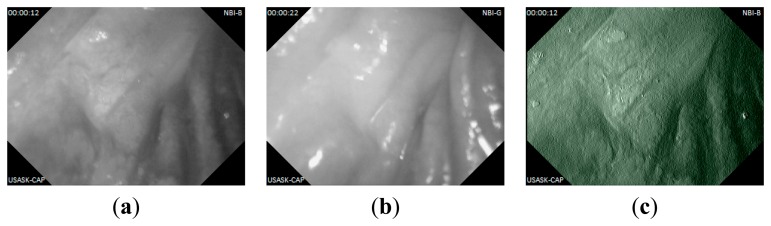
Captured NBI images from live pig's intestine: (**a**) grayscale image with blue light, CR = 84.20%; (**b**) grayscale image with green light, CR = 84.01%; (**c**) Combined pseudo color NBI image from (a) and (b).

In this experiment, anesthesia was applied to a live pig under test and the capsule prototype is place into its small intestine through surgery. The experimental setup is shown in Figure 13. The capsule captured and sent live lossless images to the outside data logger. Some captured intestine images are shown in Figures 14 and 15 with achieved CR.

The received images are clear and lossless with details of the mucosa surface of the pig intestine. Bothe the *in-vivo* and *ex-vivo* experiments indicate the effectiveness of the proposed lossless compression algorithm.

## 6. Conclusions

In this paper, a lossless image compressor tailored towards capsule endoscopy images is proposed. The input image pixels are first converted from RGB to YEF color space. The compressor then applies DPCM to calculate the difference of consecutive pixels and encodes the differences in variable length coding. The differences of luminance component are encoded in Golomb-Rice code and the differences of chrominance components are encoded in unary code. The compressor has an average compression ratio of 78% for WBI images and 84% for NBI images. It has low computational complexity and can be directly interfaced with commercial image sensors. The performance of the compressor has been validated using a miniature FPGA based capsule prototype and by performing *ex-vivo* and *in-vivo* trials with pig's intestine and live pig respectively. The results show that, the compressor consumes much lower power and area and still produces lossless intestinal images in both WLI and NBI modalities.

## Figures and Tables

**Table 1.** Average image quality index for different data bit length.

**Number of Bits (Integer + Fraction)**	**VSNR (dB)**	**Overall PSNR (dB)**	**VIF**	**SSIM**
9 (8 + 1)	75.63	58.13	0.9932	0.9989
10 (8 + 2)	97.83	98.60	0.9972	0.9997
11 (8 + 3)	∞	∞	1.0000	1.0000

**Table 2.** AVG. Absolute difference in consecutive pixel values.

	**Standard Images**		**CE Image**	
	
	**Mandrill**	**Lena**	**WLI**	**NBI**
*dY*	15.23	8.28	4.21	6.12
*dE*	1.56	0.97	0.19	0.21
*dF*	2.99	1.30	0.19	0.13

**Table 3.** *K* parameter for encoding component differences.

**Mode**	***dY***	***dE***	***dF***
WLI	2 (Golomb-Rice)	0 (unary)	0 (unary)
NBI	3 (Golomb-Rice)	Not sampled	

**Table 4.** Compression ratio of the proposed algorithm for WLI images.

**Image Description**		**Number of Images/Frames**	**Avg. CR %**
Images from Larynx to Anus of GI tract		100	74.7
Capsule endoscopy video frames	Video -1	99	80.6
	Video -2	97	80.5
	Video -3	97	80.1
	Video -4	97	80.6
	Video -5	97	79.9
Images containing disease condition	Ulcer	10	76.7
	Polyp	10	76.0
	Crohn disease	10	73.5
	Cancer	10	73.8

**Table 5.** Compression ratio of the proposed algorithm for NBI images.

**Image Description**		**Number of Images/Frames**	**Avg. CR %**
Images from different positions of GI tract		15	82.4
Capsule endoscopy video frames	Video -1	889	85.3
	Video -2	322	84.8
Images containing disease condition	Barrets oesophagus	5	82.4
	Oesophageal glycogenic acanthosis	5	83.0

**Table 6.** Compression ratio (CR %) at different bit precisions.

**No. of Bits for Fraction (After Variable Length Code for Integer)**	**WLI**	**NBI**
1	70.2	82.3
2	60.0	78.8
3	50.0	75.4

**Table 7.** Compression with other compression algorithms.

**Work**	**Type**	**Color Space**	**Algorithm**	**Avg. CR %**		**PSNR (dB)**
				**WLI**	**NBI**	
Wahid [8]	Lossy	RGB	DCT	87.1	-	32.9
Turcza [9]		RGB	DCT	96.8	-	36.5
Lin [10]		RGB	DCT with LZ77	79.6	-	32.5
Dung [13]		RGB	H.264 based DCT	82.0	-	36.2
Li [14]		RGB	DCT	75.4	-	47.7
Lin [12]		RGB	DCT with LZ77	82.3	-	40.7
Chen [24]		RGB	Modified JPEG-LS	56.7		46.4
Turcza [15]		YC_u_C_v_	DCT with Golomb-Rice	91.2	-	35.7
Khan [20]	Lossless	YUV	LPC with Golomb-Rice & Uniary	73.0	-	∞ *(Lossless)*
JPEG-LS [21]		RGB	Prediction on edge detection & Golomb-Rice	57.9	-	
Proposed		YEF	DPCM with Golomb-Rice& unary	78.0	84.0	

**Table 8.** FPGA Synthesis results of the compressor.

**Resources**	**Usage**
Number of registers	77
Number of logic cells	226
Number of block RAMs	0
Power consumption in FPGA at 2 FPS	1.63 mW

**Table 9.** Comparison of compressor with other works.

**Ref.**	**Design Platform**	**Power (mW)**	**Buffer Memory**	**FPS**	**Mode**	**Compression**	**Validated by *In-Vivo* Trial?**
[10]	ASIC 180 nm	14.92	Yes	-	WLI	Lossy	No
[24]	ASIC 180 nm	0.80	2.19 KB	2	WLI	Lossy	No
[31]	FPGA 65nm	10 ^1^	Yes	19	WLI	Lossy	No
[15]	FPGA 65nm	7 ^1^	84Kb	24	WLI	Lossy	No
Proposed	FPGA 65nm	1.63	No	50 ^2^	WLI and NBI	Lossless	Yes

1 Power of the FPGA core, only compressor power not reported;

2 Using 20 Mbps application throughput RF transceiver [32].
